# Dr. Harrison, on Ginger in Rheumatic Gout

**Published:** 1808-06

**Authors:** J. E. Harrison

**Affiliations:** Thornbury 11 Miles from Bristol


### *To the Editors of the Medical and Physical Journal.*

Gentlemen,

I Request the favour of you to publish, if you deem it worthy of notice, the following singular effects of ginger, in a case which happened to myself, on the 9th of March last. Being very much engaged in a damp room without a fire, the thermometer in my front room, situated nearly S. W. at 40°: barometer 30 inch 4. 10; hygrometer 2 inch 9. 10. I was seized with the rheumatic gout in my stomach and bowels; it began with an acute pain near the os pubis; presently the colon was as severely affected, then the small intestines, ascending rapidly into the stomach, with such prostration of strength and faintness, that I never before experienced. Assured that it was the rheumatic gout, to which I am very subject, and some sago happening to be ready boiled, I took almost a pint of it, with one large wine glass full of brandy, and a heaped table spoonfull of very fine powdered ginger. In less than an hour I felt a severe heat in my stomach, with an increase of pain, but it now *descended* through the intestinal canal to the sigmoid sigmoid flexure of the colon, and there continued almost a quarter of an hour, and then gradually declined, leaving me entirely free from pain. At bed-time, about ten, I took five drops of tincture of opium in a little brandy and water, to which I am accustomed, and had a good night's rest. In the morning, between seven and eight, I awaked well; my urine was rather high coloured and in small quantity, but deposited no sediment; I felt a little dysury, which I imputed to the effects of so large a dose of ginger.

I am, &c.

J. E. HARRISON, M. D.

*Thornbury* 11 *Miles from Bristol*,
*May* 3, 1808.

P. S. If consistent with your work, I wish my best thanks to be given to Mr. Roberton, surgeon, Edinburgh, for his excellent observations on cantharides in fluor albus. This valuable remedy has been the means of saving a woman of great respectability in South Wales, whose mother died of a cancer of the uterus. She had taken the best medical advice in London without success. I had her under my care about 18 months in all. She acknowledges that I have saved her life, but it is to Mr. Roberton she is most indebted. The case, God permitting, I will send in a short time, with some liver cases, and other complaints depending on that viscus, and the method I use in the cure of dysentery, in which I have been very successful.

